# Anticancer Activity of the Marine Triterpene Glycoside Cucumarioside A_2_-2 in Human Prostate Cancer Cells

**DOI:** 10.3390/md22010020

**Published:** 2023-12-28

**Authors:** Ekaterina S. Menchinskaya, Sergey A. Dyshlovoy, Simone Venz, Christine Jacobsen, Jessica Hauschild, Tina Rohlfing, Aleksandra S. Silchenko, Sergey A. Avilov, Stefan Balabanov, Carsten Bokemeyer, Dmitry L. Aminin, Gunhild von Amsberg, Friedemann Honecker

**Affiliations:** 1 Department of Oncology, Hematology and Bone Marrow Transplantation with Section Pneumology, Hubertus Wald Tumorzentrum—University Cancer Center Hamburg (UCCH), University Medical Center Hamburg-Eppendorf, 20251 Hamburg, Germany; christine.jacobsen@bnitm.de (C.J.); j.hauschild@uke.de (J.H.); t.rohlfing@uke.de (T.R.); c.bokemeyer@uke.de (C.B.); g.von-amsberg@uke.de (G.v.A.); friedemann.honecker@tbz-ost.ch (F.H.); 2 G.B. Elyakov Pacific Institute of Bioorganic Chemistry, Far Eastern Branch of the Russian Academy of Sciences, 159 Prospect 100-letiya Vladivostoka, Vladivostok 690022, Russia; silchenko_als@piboc.dvo.ru (A.S.S.); avilov_sa@piboc.dvo.ru (S.A.A.); daminin@piboc.dvo.ru (D.L.A.); 3 Department of Medical Biochemistry and Molecular Biology, University of Greifswald, 17475 Greifswald, Germany; simone.venz@med.uni-greifswald.de; 4 Division of Hematology, University Hospital Zurich, 8091 Zurich, Switzerland; stefan.balabanov@usz.ch; 5 Department of Biomedical Science and Environmental Biology, Kaohsiung Medical University, No. 100, Shin-Chuan 1st Road, Sanmin District, Kaohsiung City 80708, Taiwan; 6 Martini-Klinik, Prostate Cancer Center, University Hospital Hamburg-Eppendorf, 20251 Hamburg, Germany; 7 Tumor and Breast Center Eastern Switzerland, 9016 St. Gallen, Switzerland

**Keywords:** cucumarioside A_2_-2, castration-resistant prostate cancer, PC-3 cells, apoptosis, anti-metastatic activity, proteomics

## Abstract

Despite recent advances in the treatment of metastatic castration-resistant prostate cancer (CRPC), treatment is inevitably hampered by the development of drug resistance. Thus, new drugs are urgently needed. We investigated the efficacy, toxicity, and mechanism of action of the marine triterpene glycoside cucumarioside A_2_-2 (CA_2_-2) using an in vitro CRPC model. CA_2_-2 induced a G_2_/M-phase cell cycle arrest in human prostate cancer PC-3 cells and caspase-dependent apoptosis executed via an intrinsic pathway. Additionally, the drug inhibited the formation and growth of CRPC cell colonies at low micromolar concentrations. A global proteome analysis performed using the 2D-PAGE technique, followed by MALDI-MS and bioinformatical evaluation, revealed alterations in the proteins involved in cellular processes such as metastatic potential, invasion, and apoptosis. Among others, the regulation of keratin 81, CrkII, IL-1β, and cathepsin B could be identified by our proteomics approach. The effects were validated on the protein level by a 2D Western blotting analysis. Our results demonstrate the promising anticancer activity of CA_2_-2 in a prostate cancer model and provide insights on the underlying mode of action.

## 1. Introduction

Prostate cancer is the most common cancer in men aged 50+ with more than 1.3 million new cases annually, and is among the five deadliest cancers worldwide [1]. At an early stage, prostate cancer is often asymptomatic and, depending on tumor biology, can be managed by active surveillance of the patient. However, at later stages, so-called aggressive variants of prostate cancer (AVPCs) appear. AVPCs are resistant to various anticancer treatments and show an aggressive, metastases-prone phenotype [2]. Castration-resistant prostate cancer (CRPC), which is a subtype of AVPCs, is characterized by resistance to hormonal therapy, which still is the mainstay of treatment of systemic disease [3]. Despite intensive research in this area and the active search for new antitumor agents, effective ways to suppress the growth and development of CRPC tumors are still urgently needed.

Bioactive compounds derived from marine organisms are a valuable source of new anticancer agents [4]. Additionally, for many substances with established structures, previously unknown biological activities and the identification of the underlying mode of action are reported [5]. In vitro and in vivo studies of the chemo-preventive and anticancer properties of compounds isolated from fish, mollusks, starfish, and sea cucumbers indicate their great therapeutical potential [6]. 

Triterpene glycosides, produced by holothurians (also referred to as sea cucumbers), are a group of small molecules with a wide range of biological activities. Among others, they are known to be cytotoxic and ichthyotoxic, as well as antimicrobially, antivirally, radio-protectively, and immunomodulatorily active [7]. Using triterpene glycosides from sea cucumbers, several pharmacological preparations, biological additives, functional nutrition products, and cosmetics have already been developed and patented [8].

Of particular interest are the antitumor properties of these compounds. It has previously been described that holothurians’ triterpene glycosides inhibit the proliferation of human tumor cells of various origins, including cervical and lung carcinoma, liver and stomach cancer, breast and ovarian cancer, malignant melanoma, and other types [9,10,11,12,13,14,15,16]. It is also known that triterpenoids (specifically triterpene saponins of plant origin) can effectively inhibit the division of tumor cells and suppress the growth of hormone-dependent tumors [17]. However, only little is known about the antitumor activity of marine triterpene glycosides in prostate cancer. It has been reported that the triterpene saponins cercodemasoides A–E originating from the sea cucumber *Cercodemas anceps*, stichorrenosides A–D from the sea cucumber *Stichopus horrens*, and triterpene tetraglycosides from *Stichopus herrmanni*, as well as echinoside A from *Pearsonothuria graeffei*, exhibit strong cytotoxic activity at micromolar concentrations on prostate cancer models both in vitro and in vivo [18,19]. Furthermore, it could be shown that frondoside A isolated from the sea cucumber *Cucumaria okhotensis* inhibits the proliferation and formation of colonies of metastatic castration-resistant prostate cancer cells by inhibition cell cycle progression, induction of apoptosis, and inhibition of autophagy [20,21]. Additionally, in vivo, this compound inhibited the tumor growth of PC-3 and DU145 cells with a significant reduction in lung metastasis, as well as circulating tumor cells in the peripheral blood in a mouse model [20].

Cucumarioside A_2_-2 (CA_2_-2) is a triterpene glycoside which was initially isolated from the sea cucumber *Cucumaria japonica*. Our group has previously shown that CA_2_-2 is able to suppress the growth of mouse Ehrlich carcinoma cells in vitro and in vivo, can block DNA biosynthesis in the S-phase, induces apoptosis in a caspase-dependent manner, bypassing the activation of the p53-dependent segment, and causes apoptotic necrosis and mitoptosis [22,23,24,25,26]. This glycoside overcomes the multidrug resistance of tumor cells and promotes the intracellular accumulation of certain chemotherapeutic agents [27,28]. In the work presented here, we set out to elucidate the molecular mechanisms of the observed antitumor effects of CA_2_-2 using human CRPC PC-3 cells. We used a global proteomics screening approach based on 2D-PAGE electrophoresis followed by MALDI-MS and subsequent bioinformatical data analysis to identify proteins showing differential regulation upon CA_2_-2 treatment in these prostate cancer cells.

## 2. Results

### 2.1. CA_2_-2 Inhibits Prostate Cancer Cell Viability

**Figure 1:**
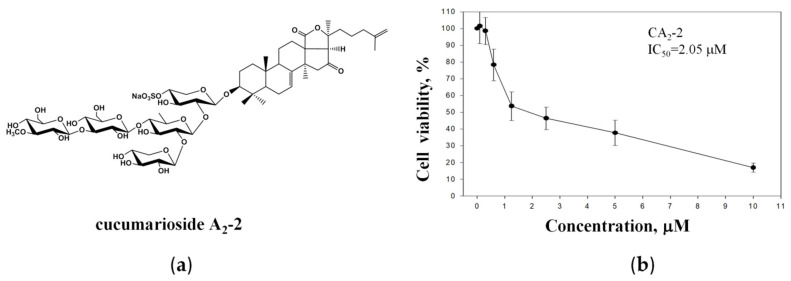
Chemical structure of CA_2_-2 (**a**). Viability of PC-3 cells treated with CA_2_-2 for 48 h and evaluated using the MTT assay (**b**).

The anticancer activity of the triterpene glycoside CA_2_-2 (Figure 1a) was assessed in metastatic castration-resistant prostate cancer PC-3 cells, known to be resistant to both anti-hormonal as well as cytotoxic agents [2]. Experiments revealed a concentration-dependent cytotoxic effect of CA_2_-2 at micromolar concentrations with an IC_50_ of 2.05 μM (Figure 1b).

### 2.2. CA_2_-2 Induces Apoptosis and Inhibits Colony Formation in PC-3 Prostate Cancer Cells

**Figure 2:**
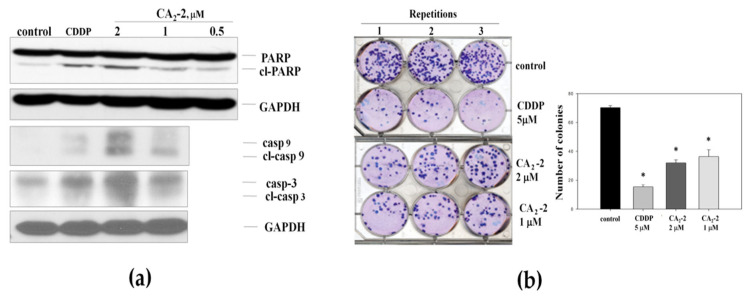
Cleavage of caspase-3, -9, and PARP-1 in PC-3 cells treated with CA_2_-2 for 48 h. Cisplatin (CDDP) was used as a positive control (**a**). Effect of triterpene glycoside of CA_2_-2 on colony formation of PC-3 tumor cells. Cisplatin (CDDP) was used as a positive control (**b**). Incubation time in either experiment was 48 h. All experiments were performed in triplicates. * *p* ≤ 0.05.

As the next step, we analyzed the ability of CA_2_-2 to induce apoptosis in PC-3 cells. We investigated a cleavage of poly (ADP-ribose) polymerase 1 (PARP-1) and a cleavage of caspases-3 and -9 as markers of apoptotic cell death (Figure 2a). Following 48 h of treatment, a dose-dependent cleavage of PARP-1 (89 kDa fragment) was observed in PC-3 tumor cells (Figure 2a). In addition, CA_2_-2 activated caspase-3 and caspase-9 at concentrations of 1 and 2 μM after 48 h of treatment. Figure 2a shows the increased expression of cleaved caspase-3 and caspase-9 compared to the control, indicating the induction of these enzymes by the triterpene glycoside.

The effect of CA_2_-2 on the ability of PC-3 cells to form colonies was studied using an in vitro colony formation assay showing the ability of single cells to form colonies. Thus, this assay can be viewed as an in vitro testing of the in vivo anti-metastatic activity of a substance [29].

Figure 2b represents images of six-well plates with cancer cell colonies stained with Giemsa dye. For the experiments, we used different concentrations for CA_2_-2, including the IC_50_ of 2 μM. Cisplatin (CDDP) at a concentration of 5 μM was used as a positive control [30], strongly suppressing the growth of PC-3 cells colonies. We found that CA_2_-2 also effectively inhibits the formation and growth of tumor cell colonies. A 30% reduction of cell colony growth was observed when CA_2_-2 was applied at concentrations of 1 to 2 μM (Figure 2b).

### 2.3. CA_2_-2 Arrests Cell Cycle of Prostate Cancer Cell 

The effects of CA_2_-2 on cell cycle progression were evaluated using flow cytometry. In this assay, anisomycin was used as a positive control, being able to induce apoptosis and increasing the fraction of aneuploid cells containing fragmented DNA (appearing as sub-G_0_ phase) [31,32,33,34].

**Figure 3:**
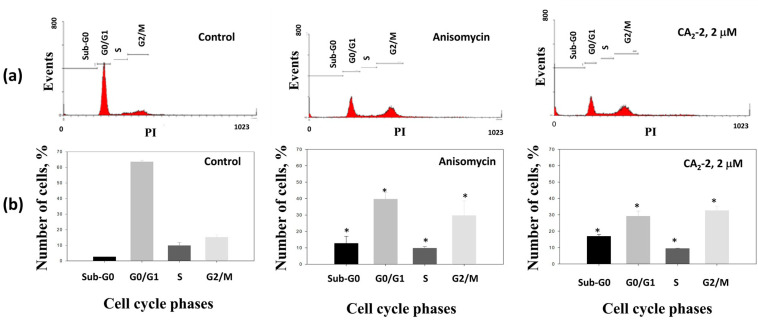
Distribution (**a**) and quantification (**b**) of the fractions of cells in different phases of the cell cycle depending on drug treatment for 48 h with either anisomycin (1 μM) as a positive control, or CA_2_-2 (2 μM). * *p* ≤ 0.05.

The incubation of PC-3 cells with the CA_2_-2 led to an increased number of cells containing fragmented DNA, suggesting the induction of apoptosis. The accumulation of cells in the mitotic phase (G_2_/M) indicates partial blocking of the cell cycle progression (Figure 3a, Table 1), whereas the number of cells in the G_0_/G_1_ phase was reduced.

Thus, the triterpene glycoside CA_2_-2 significantly increases the number of apoptotic cells, and simultaneously inhibits the cell cycle in the mitotic phase (G_2_/M) in PC-3 cells.

### 2.4. CA_2_-2 Regulates Expression of Proteins Involved in Growth, Migration, Invasion, and Cell Death in Prostate Cancer Cells 

#### 2.4.1. Proteomics Analysis of Proteins Using 2D-PAGE, MALDI-MS, and Bioinformatic Analysis

A global proteomics approach is a useful tool to identify drug targets on the protein level in cancer cells. The use of 2D electrophoresis, followed by mass spectrometry, helps to elucidate regulated protein spots and, thus, the effects of biologically active compounds on a molecular level. 

**Figure 4:**
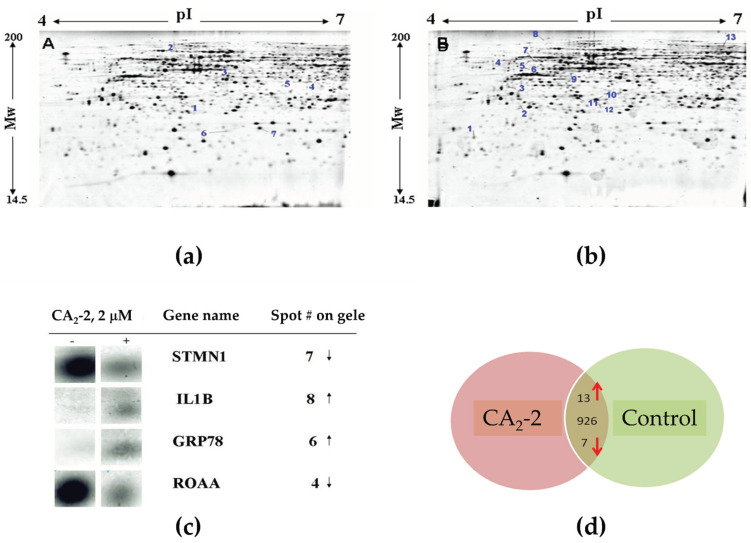
Images of 2D gel electrophoresis of non-treated (control) PC-3 cell lysates (**a**) versus lysates of PC-3 cells incubated with 2 µM of CA_2_-2 for 48 h (**b**). Spots with down-regulation upon treatment are indicated on gel A, while up-regulated spots are indicated on gel B. Magnified images of spots on a 2D gel corresponding to representative target proteins regulated by CA_2_-2 are shown in (**c**). The “−” sign in the figure denotes corresponding spots depicted for gel A (control cells), whereas “+” indicates spots depicted for gel B. Summarized information on proteins regulated in PC-3 cells is graphically shown in (**d**). Numbers indicate the total number of protein spots identified on 2D gels in the middle; arrows indicate the direction of expression of differentially regulated spots.

Hence, we performed an analysis of proteins after the treatment of PC-3 cells with CA_2_-2. Tumor cells were incubated with the compound for 48 h, followed by protein isolation and separation by two-dimensional gel electrophoresis. Figure 4 represents images of the 2D-PAGE gels from control versus CA_2_-2-treated cells. Proteins showing both down-regulation (Figure 4a) or up-regulation (Figure 4b) upon treatment were identified. A differential image analysis revealed a total number of 946 detected protein spots. In CA_2_-2-treated cells, the expression of 20 protein spots was significantly altered by the treatment (0.5 ≥ fold change ≥ 2; *p* < 0.05). Figure 4c shows representative magnified images of the protein spots significantly affected by the treatment. Among them, 13 protein spots were up-regulated, and 7 were down-regulated following exposure to CA_2_-2 (Table 2, Figure 4d). 

**Figure 5:**
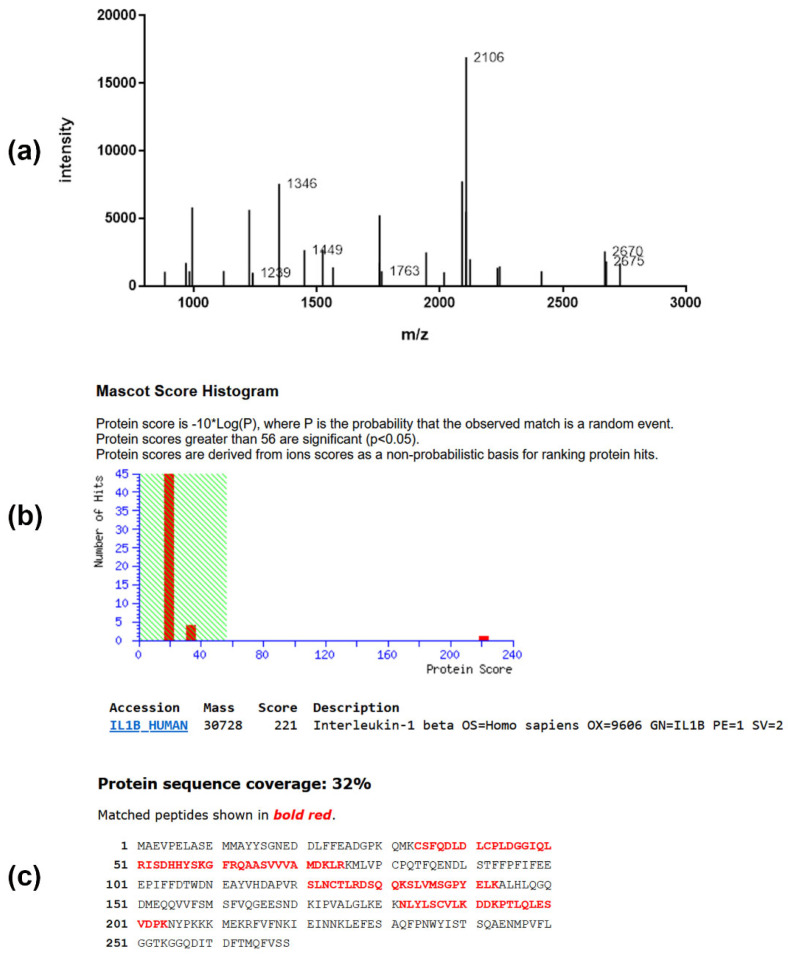
Peptide mass fingerprint spectrum (**a**), MOWSE score (**b**), and sequence coverage (**c**) of protein IL-1β of the gel derived from PC-3 cells. (**a**) MALDI mass spectrum obtained from peptide mixture derived from in-gel digestion of protein. (**b**) Database search result of protein revealed the presence of IL1B_HUMAN gene product in this spot. Identification scores greater than 56 (shaded area) are regarded as significant (*p* < 0.05). (**c**) The database entry for the protein sequence is depicted in the single letter code. The sequence stretches that are covered by peptide ion signals (81% sequence coverage) in the mass spectrum are shown in bold red.

All significantly regulated spots were subjected to peptide mass fingerprint analysis, followed by protein identification. A typical example for the identification of a protein of interest from PC-3 cells is presented in Figure 5 by the identification of IL1B (Interleukin-1β). The expression of this protein was found to be significantly changed upon treatment with triterpene glycoside (see Table 2). 

The identified proteins regulated by CA_2_-2 could be assigned to different groups according to their supposed biological functions (Table 2). Most of these proteins identified play an important role in the functioning of tumor cells, including some well-established tumor markers. Interestingly, the largest group of proteins regulated by CA_2_-2 could be assigned to proteins involved in the structural organization of the cytoskeleton, as well as in cell movement and proliferation (32%). Another frequent finding was proteins playing an important role in the functioning of the cell nucleus (21%).

#### 2.4.2. Verification of Protein Expression by Western Blotting and 2D Western Blotting

A proteomics analysis identified 20 proteins whose expression was significantly altered following treatment with CA_2_-2. To verify the proteomics data, we selected four proteins and analyzed their expression using 1D and 2D Western blotting. The expressions of keratin-81, Crk II, interleukin-1β, and cathepsin B were examined.

**Figure 6:**
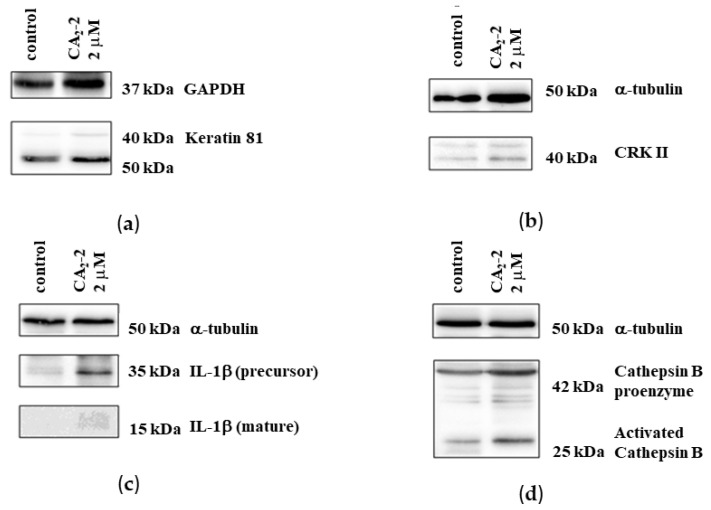
Immunoblots of keratin 81 (**a**), CrkII (**b**), IL-1β (**c**), and cathepsin B (**d**) upon treatment of PC-3 cells for 48 h with CA_2_-2.

**Figure 7:**
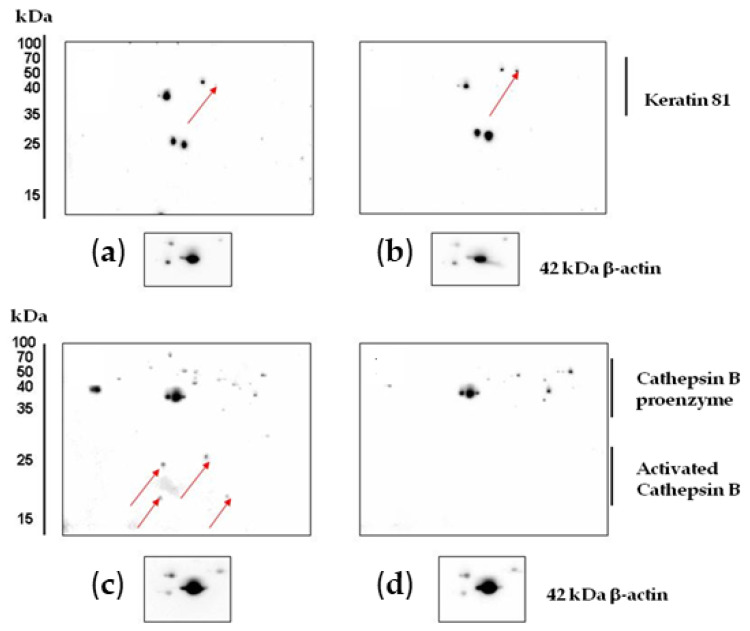
Results of 2D Western blot analysis of changes in the expression of keratin 81 in control (**a**) and CA_2_-2-treated cells (**b**). Cathepsin B control (**c**) and CA_2_-2-treated PC-3 cells (**d**). CA_2_-2 was used at 2 μM for 48 h. The arrows on the gels indicate the spots corresponding to the studied proteins.

Experiments on protein verification by immunoblotting showed that treatment with CA_2_-2 resulted in a significant increase in the expression of both interleukin-1β isoforms (Figure 6c). However, at the same time, the expression of the keratin 81 protein remained at the control level (Figure 6a). Therefore, a 2D Western blot of keratin 81 was performed, showing a significant increase in the expression of a specific isoform of keratin 81 compared to the controls (Figure 7a,b). The expression of the CRK II protein was found to be increased when cells were incubated with CA_2_-2 (Figure 6b). 

Taken together, we could demonstrate that CA_2_-2 causes changes in the expression of a number of proteins that play roles in the functioning of tumor cells. These molecular/submolecular processes are involved in a broad spectrum of biological activities, including the mechanisms of cancer cell development and division, tumor invasion, and the programmed death of tumor cells. Therefore, the data generated using a global proteomics approach are in good agreement with the observation that the antitumor activity of triterpene glycosides is exerted by their ability to abolish tumor cell proliferation, block the cell cycle, and induce tumor cell apoptosis.

## 3. Discussion

Triterpene glycosides of holothurians are characterized by a wide spectrum of dose-dependent biological activities. Used at high concentrations, triterpene glycosides are known to exhibit membranolytic properties leading to hemolysis, cytotoxicity, and antimicrobial and embryotoxic activity [7]. On the other hand, at low nanomolar concentrations, these compounds can demonstrate immunostimulatory activity by the activation of immunocompetent cells [22], induction of apoptosis in tumor cells, and blockade of the cell cycle and proliferation [23].

Here, we present, for the first time, that the triterpene glycoside CA_2_-2 induces apoptosis in drug-resistant human prostate cancer PC-3 cells. Apoptosis upon treatment with CA_2_-2 is characterized by the activation of proapoptotic caspases (caspase-3 and -9) and by the appearance of a cleavage product of the substrate of activated caspases, i.e. cleaved PARP-1 protein. The effect of cucumarioside A_2_-2 was accompanied by a significant increase in the number of aneuploid cells containing fragmented DNA and appearing in the sub-G_0_ phase, as well as a blockade of the cell cycle in the G_2_/M mitosis phase. Moreover, CA_2_-2 is able to effectively abrogate the formation and growth of colonies of human tumor PC-3 cells.

Searching for the mode of action and involved pathways, we used a global proteomics screening approach. Protein groups found to be differentially expressed upon exposure of PC-3 cells to CA_2_-2 can be classified as important regulators of cell metabolism, motility, and division, as well as the structural and functional organization of the cytoskeleton. The identified direct or indirect protein targets, including stathmin, kinesin, caldesmon, clathrin, CRABP2, nucleophosmin, lamin-B1, nesprin-1, CRK II, and others, play a key role in the functioning of tumor cells and are closely associated with mechanisms of the development and regulation of mitosis, tumor cell proliferation, cell migration, and tumor invasion, and control of programmed death. 

It was found that CA_2_-2 increases the expression of GRP78 and IL-1β. Those proteins play complex and multiple roles in the development and progression of tumors. Thus, some studies describe that the GRP78 protein promotes the growth and invasion of tumor cells [35,36]. Other reports show that the high expression of GRP78 promotes the effectiveness of chemotherapy and improves the disease-free survival of patients [37]. 

Speaking about the role of IL-1β, it should be noted that it plays a role in many processes in the human body. In oncogenesis, both positive and negative functions of this protein have been described, depending on the type of tumor. A high IL-1β expression in prostate cancer predicts good treatment prognosis and better progression-free survival [38]. And, when LNCaP prostate cancer cells are co-cultured with fibroblasts, the high expression of IL-1β is antiproliferative [39]. The first results of the effect of IL-1b on metastasis were shown 30 years ago. Thus, fibrosarcoma cells that overexpress IL-1β have been shown to have increased invasive potential [40]. Recently, the inhibition of IL-1β was found to reduce the metastatic potential of murine prostate cancer cells while its overexpression was increased [41]. These new insights are a first step to understanding the underlying molecular mechanisms of the observed anticancer activities hiding in holothurian glycosides.

It is well-known that some marine triterpene glycosides exhibit pronounced anticancer effects by direct interaction with tumor cells in the sub-cytotoxic concentration range [6,8]. However, we are only beginning to understand the molecular mechanisms and signaling pathways involved in their antitumoral activity. We postulate that triterpene glycosides suppress the function of tumor cells by their ability to induce caspase-dependent or -independent apoptosis, arrest the cell cycle in certain phases, and control the expression of the nuclear factor NF-κB, as well as regulate the expression of cellular receptors and enzymes involved in carcinogenesis, such as EGFR, Akt, ERK, FAK, MMP-9, and some others [5].

So far, to our knowledge, only for one triterpene glycoside, frondoside A, several target proteins were identified [20]. A global proteome analysis and bioinformatic approach revealed the regulation of proteins involved in the formation of metastases, tumor cell invasion, and apoptosis, like keratin 81, CrkII, IL-1β, and cathepsin B [20]. Comparing the results of the proteomics analyses of both frondoside A and CA_2_-2, we found only five proteins that are equally regulated by these proteins. The proteins most significantly regulated by CA_2_-2 were recognized as proteins encoded by the IL1B, KRT81, and CRK II genes (up-regulated), as well as proteins encoded by the CATB and ROAA genes (down-regulated). A literature search for the functional activity of these proteins in databases revealed that these proteins are directly involved in the regulation of tumor cell proliferation, tumor invasion, metastasis, and apoptosis [42,43,44,45,46].

Moreover, in vivo, frondoside A inhibited the tumor growth of PC-3 and DU145 cells with a notable reduction of lung metastasis, as well as circulating tumor cells in the peripheral blood [20]. The results of the current study indicate that the mechanism of action of CA_2_-2 may be similar to those of frondoside A, making this class of chemicals highly interesting substances for drug development in oncology. 

## 4. Materials and Methods

### 4.1. Reagents and Antibodies

The marine triterpene glycoside cucucmarioside A_2_-2 was isolated from the extract of sea cucumber *Cucumaria japonica* as previously reported [47]. The purity of cucucmarioside A_2_-2 was determined by ^13^C NMR spectroscopy and ESI mass-spectrometry. 

MTT solution (Sigma-Aldrich, Burlington, MA, USA), Giemsa’s solution (Merck, Darmstadt, Germany), trypsin-EDTA (gibco^®^ Life technologies^TM^, Paisley, UK), propidium iodide solution (Sigma-Aldrich, Burlington, MA, USA), RNase (Carl Roth, Karlsruhe, Germany), CHAPS (Sigma-Aldrich, Burlington, MA, USA), 1% Pharmalyte (Sigma-Aldrich, Burlington, MA, USA), Coomassie Brilliant Blue G 250 (Thermo scientific, Rockford, IL, USA), anisomycin (Sigma-Aldrich, Burlington, MA, USA), DMEM medium (Lonza, Walkersville, MD, USA), fetal calf serum (FBS) (gibco^®^ Life technologies^TM^), penicillin-streptomycin (gibco^®^ Life technologies^TM^), polyclonal rabbit antibodies against PARP-1, titer 1:1000 (Cell signaling, Danvers, MA, USA); monoclonal rabbit antibodies against caspase-3, titer 1:1000 (Cell signaling, Danvers, MA, USA); monoclonal mouse antibodies against caspase-9, titer 1:1000 (Cell signaling, Danvers, MA, USA); and polyclonal rabbit antibodies against GAPDH (abcam, Cambridge, MA, USA) were used. Secondary goat antibodies labeled with horseradish peroxidase against rabbit, titer 1:5000 (abcam, Cambridge, MA, USA), and secondary rabbit antibodies against mouse, titer 1:10,000 (abcam, Cambridge, MA, USA) were used.

### 4.2. Cell Lines and Culture Conditions

The human prostate cancer androgen-independent cell line PC-3 was obtained from ATCC (CRL-1435 ™, Manassas, VA, USA). PC-3 cells were cultured as a monolayer under standard conditions (37 °C, 5% CO_2_) in DMEM medium supplemented with 10% fetal bovine serum (FBS) and 1% penicillin-streptomycin.

### 4.3. MTT Assay

The cytotoxic activity of CA_2_-2 was measured by the MTT method. 20 μL of a solution of the test compound of various concentrations was added to the wells of a 96-well plate to 180 μL of adherent PC3 cells (6 × 10^3^ cells/well), and then incubated for 48 h at 37 °C and 5% CO_2_. After incubation, 200 μL of the supernatant was replaced and 100 μL of pure medium and 10 μL of MTT solution (5 mg/mL in PBS) were added. They were further incubated for 4 h and 100 μL of SDS-HCl solution was added and incubated at 37 °C for 18 h. Absorbance was measured at 570 nm using an Infinite F200PRO plate reader (TECAN, Mannedorf, Switzerland). The cytotoxic activity of a substance was expressed as the IC_50_ concentration at which the metabolic activity of cells is inhibited by 50%.

### 4.4. Colony Formation Assay

PC-3 cells were seeded on small Petri dishes d = 35 mm at a concentration of 3 × 10^5^ cells in 5 mL of DMEM medium for 24 p for adhesion at 5%, CO_2_, 37 °C. Then, 50 μL of Kuk solution at various concentrations was added to the cells and placed in a CO2 incubator for 48 h. Then, the cells were treated with trypsin (0.25% trypsin in 1 mM EDTA solution) and washed with PBS by centrifugation for 5 min, 1500 rpm, and resuspended; the number of living and dead cells was counted using an automated Beckman Coulter Vi-CELL instrument (Beckman Coulter, Krefeld, Germany) and seeded into 6-well plates at a concentration of 100 live cells in 3 mL of DMEM medium. After 10 days of incubation, the medium was taken, the cells were fixed with 1 mL of 100% methanol for 25 min, washed with PBS and dried, and 1 mL of Giemsa solution was added, and then they were incubated for 25 min at room temperature and washed with dH_2_O [36]. The cells were then air dried and the number of colonies was counted. Results were expressed as the number of colonies grown per well.

### 4.5. Cell Cycle Analysis

PC-3 cells in the amount of 2 × 10^6^ cells/mL were plated on Petri dishes in complete growth medium and incubated for 24 h for adhesion (5% CO_2_, 37 °C). After that, the medium was changed, and solutions of substance in different concentrations of 100 μL were added and placed in an incubator for 48 h. Then, the cells were treated with a solution of trypsin, the total number of cells was counted, and 1 × 10^6^ cells were taken from each sample. The cell pellet was resuspended in 300 μL of cold phosphate-buffered saline (PBS), fixed in 700 μL of 100% ethanol, and left for 24 h at −20 °C. Then, the cells were washed twice from ethanol and resuspended in 200 μL of propidium iodide solution (PI, 5 μg/mL in PBS), RNase 20 μg/mL. The cells were then transferred to flow cytometry tubes and incubated for 30 min at room temperature in the dark. Before analysis, 500 μL of cold PBS was added to the cell suspension. The study was carried out using an FACScalibur flow cytometer (Becton Dickinson, USA) at 488 nm excitation in the FL2 channel. Histograms were evaluated using the WinMDI 2.9 Ink program (USA).

### 4.6. Western Blotting

For the determination of caspase-3, -9, and PARP-1 proteins by Western blotting, cells were plated on Petri dishes in the amount of 1 × 10^6^ cells in 5 mL of complete growth medium and placed in a CO_2_ incubator for 24 h for adhesion. After that, the medium was replaced with a fresh one with the addition of substance in the investigated concentrations. After 24 h or 48 h, the cells were treated with Trypsin-EDTA solution and washed with PBS three times. Next, the cell pellet was resuspended in 100 μL of lysis buffer solution (0.88% NaCl, 50 mM Tris-HCl (pH 7.6), 1% NP-40, 0.25% sodium cholate, 1 mM PFMS, 1 mM Na_3_VO_4_) and incubated 40 min on ice, after which it was left overnight at −20 °C. Lysed cells were pelleted by centrifugation for 10 min at 14000 rpm and 4 °C. Protein concentration was measured according to the Bradford method. Samples with proteins were heated to 95 °C for 5 min. The electrophoretic separation of proteins was carried out according to the Laemmli method [48] in 10% PAGE at a voltage of 80 V for the first 30 min and at 120 V for the remaining time.

After electrophoretic separation, proteins were transferred from the gel to a PVDF membrane (0.45 m, Millipore Corporation, Burlington, MA, USA) at 25 V for 1 h using a Trans-blot SD Semi-dry transfer cell (Bio-rad, USA). Before incubation with primary antibodies overnight at 4 °C, the membrane was blocked with 5% BSA solution in 0.05% TBS-Tween 20 solution for 1 h. Then, the membrane was washed from primary antibodies with TBS-Tween 20 solution 3 times for 5 min and incubated for 1 h with secondary antibodies labeled with horseradish peroxidase at room temperature. After that, 1 mL of Pierce ECL Western Blotting Substrate (Thermo scientific, Rockford, IL, USA) solutions were added to the membranes, a CL-XPosureTM Film 5 × 7 inches X-ray film (Thermo scientific, Rockford, IL, USA) was applied, and the signal was visualized on the X-ray film after its development using a developing machine (AGFA CP 1000, Mortsel, Belgium).

### 4.7. Two-Dimensional Gel Electrophoresis (2D-PAGE)

Experiments on the analysis of protein expression using the proteomics approach were carried out on PC-3 cells by two-dimensional electrophoresis (2D-PAGE) and subsequent analysis of the protein structure by mass spectrometry. The cells were seeded in flasks at a concentration of 5 × 10^6^ cells in 30 mL of complete growth medium and placed in a CO_2_ incubator for 24 h for adhesion. Then, triterpene glycoside solutions were added to the cells, and the cells were incubated for 48 h. After that, the cells were collected and washed twice by centrifugation in PBS solution. The cell pellet was resuspended in 500 μL of lysis buffer of the following composition, 9 M urea, 4% CHAPS, 1%Pharmalyte, 1% DTT, and bromophenol blue 10 μg/mL, and left overnight at −20 °C. Then, the samples were precipitated by centrifugation for 10 min, 4 °C, and 14,000 rpm, and 450 μL aliquots with 1 mg of proteins were applied to strips for isoelectric focusing (Immobiline Dry Strip pH 4–7, 24 cm, GE Healthcare Bio-Sciences AB, Uppsala, Sweden) for 24 h to rehydrate at RT. Next, the first separation was performed using a Protean IEF cell (Bio-Rad, Hercules, CA, USA). After the first separation, the strips were equilibrated in 1% DTT solution supplemented with 6 M urea, 4% SDS, 50 mM Tris-HCl pH 8.8 for 15 min, and then alkylated with 4.8% iodoacetamide solution for 15 min at RT. Then, the strips were transferred onto a 15% SDS-PAGE gel (27 cm × 21 cm × 1.5 mm) and covered with a 0.6% agarose solution. The second electrophoretic separation was carried out in SDS-buffer solution of the following composition: 1.44% glycine, 0.3% Tris base, 0.1% SDS at 20 °C for 14 h. The gels were fixed and stained with a Coomassie solution (400 mL/gel containing 0.13% Coomassie Brilliant Blue G 250, 3.6% H_2_SO_4_, 1.44 M NaOH, 20.3% CCl_3_COOH) overnight, after which the gels were washed with dH_2_O three times.

### 4.8. 2D-Gel Image Analysis and Protein Identification by Mass Spectrometry

The studied protein spots were excised from the gel and transferred to a 96-well plate. Spot matching, normalization of the digital images (based on total optical density), and gel image analysis were performed using Delta 2D 4.0 software (Department of Medical Biochemistry and Molecular Biology, University of Greifswald, Germany). Briefly, trypsin digestion, followed by transfer of aliquots of protein solutions to MALDI targets, was performed automatically on an Ettan Spot Handling Workstation (Amersham-Biosciences, Sweden) using a modified standard protocol. MALDI-MS measurements of sample measurements were performed on the 4800 MALDI-ToF/ToFTM Analyzer (Applied Biosystems, Waltham, MA, USA). Recording spectra in the reflex mode in the mass range from 0.8 to 4 kDa, with a mass change of 2 kDa. Additionally, for the five most intense peaks of the MS spectra, MALDI-MS/MS analysis was performed; after subtracting the peaks, there were background or trypsin fragments. Comparison of MS revenues and MS/MS data with usage efficiency was reported in databases using GPS Explorer, Ver. 3.6 (Applied Biosystems, Waltham, MA, USA). The SwissProt v56.1 database was used for identification.

### 4.9. Bioinformatic Analysis of Differentially Expressed Proteins

The following databases were used to search for the functions of proteins:

UniProtKB/Swiss-Prot (http://www.uniprot.org, accessed on 4 September 2023); GeneCards (http://www.genecards.org, accessed on 4 September 2023); Gene Ontology Consortium (http://www.geneontology.org, accessed on 4 September 2023); Bioinformatic Harvester (http://www.embl.de/services/bioinformatics, accessed on 4 September 2023); PubMed (http://www.ncbi.nlm.nih.gov/pubmed, accessed on 4 September 2023).

### 4.10. Mini 2D Western Blotting Analysis (2D-WB)

Two-dimensional Western blotting (2D Western blotting) was used to confirm the results of 2D-PAGE electrophoresis. Aliquots of protein extracts containing 30 μg of protein were diluted using 2D-PAGE-lysis buffer to a volume of 125 μL, and then loaded onto immobilized pH-gradient (IPG) Immobiline Dry Strip (pH 4–7, 7 cm, GE Healthcare Bio-sciences, Sweden). The IPG strips were incubated overnight at RT for rehydration. Next, the IPG strips were subjected to isoelectric focusing (IEF) using a Protean IEF cell (Bio-Rad, Germany). Separation of proteins in the first dimension (isoelectric focusing) was carried out sequentially in 4 stages: 1–30 min at 500 V; 2–5 kV/h at 5 kV (“linear focusing”); 3–5 kV/h at 5 kV (“fast focusing”); 4–2 h at 500 V. After the IEF step, the IPG strips were stored at −80 °C.

After the first separation, the strips were equilibrated in 1% DTT solution supplemented with 6 M urea, 4% SDS, 50 mM Tris-HCl pH 8.8 for 15 min, and then alkylated with 4.8% iodoacetamide solution for 15 min at RT and loaded onto SDS-polyacrylamide gels containing 15% acrylamide and prepared as described for Western blotting, but without the concentrating gel. A marker (Spectra multicolor Broad Range Protein Ladder, Fermentas, Finland) in an amount of 10 μL was applied to 0.5 cm^2^ filter paper, placed on the gel next to the IPG strip, and embedded in a 0.6% agarose solution in dH_2_O containing 0.001% (*w*/*v*) bromophenol blue as a dye. Proteins were separated in a Mini-PROTEAN 3 electrophoresis chamber (Bio-Rad, Hercules, USA) in a buffer solution for protein separation in polyacrylamide gel. Electrophoresis was carried out for ~3 h at 65 V at RT to the required degree of protein separation controlled by the marker. Subsequent transfer of proteins to the membrane, blocking of the membrane, incubation with primary and secondary antibodies, and signal detection were performed in the same way as described for one-dimensional Western blotting (see Section 4.6). After the transfer step, polyacrylamide gels were stained with Coomassie Brilliant Blue colloidal solution G-250 (20 mL/gel), followed by washing with dH_2_O as described for 2D-PAGE (see Section 4.8). After washing, the gels were scanned using a GS-800 Calibrated Densitometer (Bio-Rad, Hercules, USA). Digital images of the gels were used as a control for loading the same amount of protein for the 2D Western blot method. The relative optical density of protein spot signals was quantitatively analyzed using the Bio 1D 15.01 software (Vilber Lourmat, Eberhardzell, Germany).

### 4.11. Statistics

All data are expressed as mean ± S.E. from three or more experiments, and were statistically evaluated by Student’s *t*-test using SigmaPlot 14.0 (Jandel Scientific, San Rafael, CA, USA). Differences were considered significant when *p* ≤ 0.05. 

## 5. Conclusion

In summary, the triterpene glycoside, CA_2_-2, isolated from the sea cucumber *C. japonica*, is a small bioactive molecule capable of inducing apoptosis in tumor cells via the caspase-dependent intrinsic pathway. Furthermore, it leads to a blockade of the cell cycle in the G2/M phase. Among others, keratin 81, CrkII, IL-1β, and cathepsin C were identified as proteins directly or indirectly targeted by CA_2_-2 in human prostate cancer cells. Given the promising anticancer activity of CA_2_-2, these and other newly identified molecular targets are worth being investigated further.

## Figures and Tables

**Table 1:** Effect of CA_2_-2 on cell cycle progression of PC-3 cells. Anisomycin was used as a positive control. Treatment time was 48 h.

Substance	Cell Cycle Phases (%)			
	SubG_0_	G_0_/G_1_	S	G_2_/M
Control (untreated)	2.59 ± 0.08	63.54 ± 1.01	9.85 ± 2.05	15.22 ± 1.71
CA_2_-2 (1 μM)	8.78 ± 0.23 *	36.87 ± 1.53 *	9.22 ± 0.04	32.14 ± 0.30 *
CA_2_-2 (2 μM)	16.92 ± 1.10 *	29.19 ± 3.11 *	9.43 ± 0.20	32.56 ± 2.72 *
Anisomycin (1 μM)	12.71 ± 4.32 *	39.63 ± 4.34 *	9.81 ± 1.09	29.62 ± 9.04

* *p* ≤ 0.05 compared with control.

**Table 2:** Proteins regulated by CA_2_-2 in human PC-3 prostate cancer cells. The numbers of down-regulated proteins correspond to the numbers of spots in Figure 4a; the numbers of up-regulated proteins correspond to the numbers of spots in Figure 4b.

Spot No on Gel	Gene Name	Protein	Up/Down Regulation, Fold Change	Protein Function
**Protein metabolism, enzymatic activity**				
4	PDIA1	Protein disulfide-isomerase	2.44 **↑**	Catalyzes the formation and destruction of disulfide bonds during protein folding
1	CATB	Cathepsin B	3.2 **↓**	Takes part in apoptosis, and is a mediator of the lysosomal pathway of cell death
6	GRP78	78 kDa glucose-regulated protein	2.43 **↑**	Controls the processes of invasion, apoptosis, and inflammation
**Metabolism of carbohydrates**				
5	PGP	Phosphoglycolate phosphatase	2.12 **↓**	Takes part in the metabolism of carbohydrates
**Cytoskeletal organization, cell motility, and division**				
2	K2C1	Keratin, type II cytoskeletal 1	2.35 **↑**	Participates in the formation of intermediate filaments
11	KRT81	Keratin, type II cuticular Hb1	2.96 **↑**	Participates in the formation of intermediate filaments
10	KRT81	Keratin, type II cuticular Hb1	2.52 **↑**	Participates in the formation of intermediate filaments
7	STMN1	Stathmin	2.16 **↓**	Regulates rapid cytoskeletal remodeling in response to cell needs
5	KINH	Kinesin-1 heavy chain	2.22 **↑**	Supports mitosis, meiosis, and transport of intracellular molecules
13	CALD1	Caldesmon	2,18 **↑**	Binds calmodulin, and inhibits the ATPase activity of myosin
12	CRK II	Adapter molecule crk	2.93 **↑**	Involved in phagocytosis of apoptotic cells, and may regulate EFNA5-EPHA3 signaling
**Immune response**				
9	IL1B	Interleukin-1 beta	3.32 **↑** (precursor)	Development and regulation of the body’s defense response to a pathogen
3	IL1B	Interleukin-1 beta	3.09 **↑** (mature)	Development and regulation of the body’s defense response to a pathogen
**mRNA processing**				
4	ROAA	Heterogeneous nuclear ribonucleoprotein A/B	2.04 **↓**	Regulates the formation of telomeres and/or their stabilization, and also takes part in the control of apoptosis
8	HNRL2	Heterogeneous nuclear ribonucleoprotein U-like protein 2	2.27 **↑**	Process heteronuclear RNA into mature mRNAs, and regulates of gene expression
**Response to stress**				
3	NDRG1	Protein NDRG1	2.2 **↓**	Participates in the formation of a response to stress and hormones, and participates in cell growth and differentiation
**Structural and functional organization of the nucleus**				
6	CRABP2	Cellular retinoic acid-binding protein 2	2.16 **↓**	Is an intracellular lipid-binding protein that interacts with cyclin D
1	NPM	Nucleophosmin	2.16 **↑**	Takes part in the biogenesis of ribosomes, and the transport of proteins to the nucleus
2	LMNB1	Lamin-B1	4.01 **↓**	Performs structural functions, and takes part in the regulation of transcription
7	SYNE1	Nesprin-1	2.15 **↑**	Takes part in the nuclear organization and structural structure of the nucleus, and interacts with F-actin and with the nuclear envelope

## Data Availability

The data that support the findings of this study are available from the corresponding author upon reasonable request.
